# Errata

**Published:** 2005-07

In “Seasick Lungs: How Airborne Algal Toxins Trigger Asthma Symptoms” [ Environ Health Perspect 113:A324 (2005)], the accompanying photograph of *Karenia brevis* should have been credited to Daniel Baden/University of North Carolina at Wilmington.

“Linking Toenail Arsenic Content to Cutaneous Melanoma” [ Environ Health Perspect 113:A377 (2005)] should have clarified that Laura E. Beane Freeman received NIEHS funding while at the University of Iowa, before she joined the National Cancer Institute.

*EHP* regrets the errors.

